# Identification of Fungal Communities Associated with the Biodeterioration of Waterlogged Archeological Wood in a Han Dynasty Tomb in China

**DOI:** 10.3389/fmicb.2017.01633

**Published:** 2017-08-24

**Authors:** Zijun Liu, Yu Wang, Xiaoxuan Pan, Qinya Ge, Qinglin Ma, Qiang Li, Tongtong Fu, Cuiting Hu, Xudong Zhu, Jiao Pan

**Affiliations:** ^1^Key Laboratory of Molecular Microbiology and Technology for Ministry of Education, Department of Microbiology, College of Life Sciences, Nankai University Tianjin, China; ^2^Chinese Academy of Cultural Heritage Beijing, China; ^3^Laboratory of Cultural Relics Conservation Materials, Department of Chemistry, Zhejiang University Hangzhou, China

**Keywords:** Han Dynasty tomb, *huangchang ticou*, biodeterioration, high-throughput sequencing, fungal community, *Hypochnicium* sp.

## Abstract

The Mausoleum of the Dingtao King (termed ‘M2’) is a large-scale *huangchang ticou* tomb that dates to the Western Han Dynasty (206 B.C.–25 A.D.). It is the highest-ranking Han Dynasty tomb discovered to date. However, biodeterioration on the surface of the tomb M2 is causing severe damage to its wooden materials. The aim of the present study was to give insight into the fungal communities colonized the wooden tomb. For this purpose, seven samples were collected from different sections of the tomb M2 which exhibited obvious biodeterioration in the form of white spots. Microbial structures associated with the white spots were observed with scanning electron microscopy. Fungal community structures were assessed for seven samples via a combination of high-throughput sequencing and culture-dependent techniques. Sequencing analyses identified 114 total genera that belonged to five fungal phyla. *Hypochnicium* was the most abundant genus across all samples and accounted for 98.61–99.45% of the total community composition. Further, *Hypochnicium* sp. and *Mortierella* sp. cultures were successfully isolated from the tomb samples, and were distinguished as *Hypochnicium* sp. WY-DT1 and *Mortierella* sp. NK-DT1, respectively. Cultivation-dependent experiments indicated that the dominant member, *Hypochnicium* sp. WY- DT1, could grow at low temperatures and significantly degraded cellulose and lignin. Thus, our results taken together suggest that this fungal strain must be regarded as a serious threat to the preservation of the wooden tomb M2. The results reported here are useful for informing future contamination mitigation efforts for the tomb M2 as well as other similar cultural artifacts.

## Introduction

**FIGURE 1:**
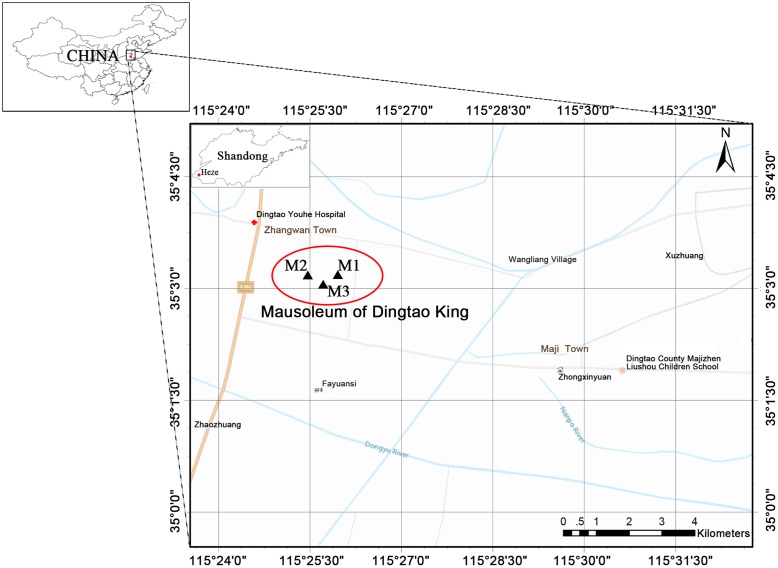
The location of the Dingtao King’s Mausoleum in Shandong Heze, China. The mausoleum consists of three tombs: M1, M2, and M3. The M1 and M3 tombs are stone-chamber tombs, while the M2 is a large-scale *huangchang ticou* tomb. The map was drawn using ArcGIS v.10.2 (www.esri.com/arcgis).

The ‘M2’ Mausoleum of the Dingtao King dates to the Western Han Dynasty and is located 200 m northwest of the Lijiacun Village in the Maji Township of the Dingtao District of Shandong Heze City (**Figure 1**). The site has been referred to as one of China’s 10 most important archeological discoveries due to its value toward research on the imperial mausoleums of the Western Han Dynasty. The M2 tomb represents the best-preserved large-scale *huangchang ticou* tomb that has been found in China and is very valuable for research on the *huangchang ticou* (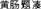) burial system (Cui et al., 2013).

*Huangchang ticou* was a burial form in ancient China that emerged in the spring and autumn period (770–476 B.C.) and became prevalent during the Western Han Dynasty (206 B.C.–A.D. 25). *Huangchang ticou* consist of piled wooden walls of cypress heartwood timbers that encompass the inner and outer coffins of the tomb occupant. The burial form material is usually cypress with bark that has been removed (Cui et al., 2013). *Huangchang* indicates the color and shape of the material and refers to the cypress heartwood timbers that appear faint yellow. *Ticou* refers to the shape and structure of the piled-up material, which largely comprises wooden cross-sections of yellow cypress that face the inside and outside of the chambers and form the *ticou* wall (Huang, 1998). *Huangchang ticou* was a burial standard for emperors like jade shrouds, catalpa coffins, burial lounges, and exterior burial pits for carriages or kitchen wares and grains. Later, the standard was extended to empresses, imperial concubines, favored ministers, and vassal kings and queens. Currently, there are about 10 *huangchang ticou* tombs from the Han Dynasty that are being excavated in China.

The Mausoleum of the Dingtao King was excavated in October of 2010. The tomb resembles the Chinese character “
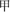
” and its roof and ticou walls are sealed with bricks. The whole chamber is square with 22.8 m long walls. The wooden coffin chambers exhibit a large-scale huangchang ticou architectural complex that consists of four main parts—a front chamber, a central chamber, a rear chamber, and its eight side chambers. The structure also consists of 12 outer storage chambers, corridors, passages connecting the main chambers, four doorways, and ticou walls (**Supplementary Figure S1**). As a result of underground water intrusion, the tomb is highly waterlogged, and has consequently remained well-conserved.

**FIGURE 2:**
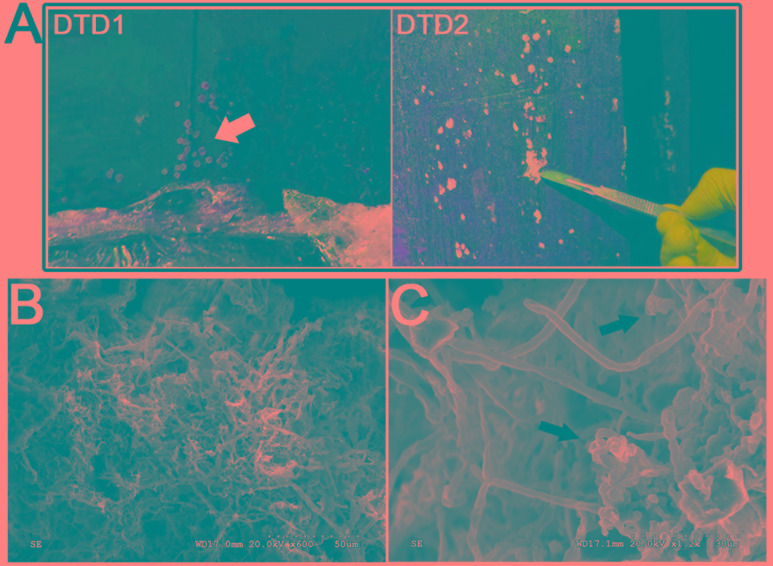
Morphological observations of the heavy fungal colonization of the wooden tomb. **(A)** Sample DTD1, which exhibited a sporocarp appearance was used for microbial isolation and identification. Sample DTD2 was collected from white spots on the surface of the tomb. **(B,C)** Scanning electron micrograph of a white spot. Visualization of fungal hyphae and spores which appear ellipsoidal and spherical. The small dots indicate the bar.

After its discovery, fungal mycelia were observed on the surface of the tomb M2, as noted by white spots that were first observed in September 2012 (**Figure 2A** and **Supplementary Figure S2**). Since the onset of our investigation into the biodeterioration of this wooden tomb in March 2015, the white spots have spread to every chamber. In addition to the white spots, insects including fungus gnats, spiders, and millipedes have been found in the tomb that may also contribute to the propagation of the white spots. Microorganisms, and especially filamentous fungi, are able to cause biodeterioration of archeological wood (Irbe et al., 2012; Ortiz et al., 2014; Gutarowska et al., 2015; Piñar et al., 2016). Thus, the biodeterioration of the wooden tomb due to microorganisms that can degrade lignin and cellulose must be investigated with urgency.

In order to inform the preservation of the wooden tomb M2, the aims of our study were to characterize the fungal communities of the white spots. The spots were analyzed through a combination of scanning electron microscopy (SEM) and high-throughput sequencing. Further, we isolated the predominant fungal populations and assessed their ability to degrade lignin and cellulose. Finally, the results of this study are summarized with regards to conservation recommendations and biodeterioration control for the wooden tomb.

## Materials and Methods

### Sample Collection

The total surface area of the tomb is about 3,000 m^2^ and the total volume of the wood that was used is ∼2,200 m^3^. The primary construction material was cypress and pine. The temperature inside the tomb remains between 10 and 16°C year-round. The mound of the tomb is ∼11 m deep and is affected by Yellow River silting.

Wood samples DTD1–DTD7 were collected from different locations in the tomb and the sampling sites are shown in **Figure 2A** and **Supplementary Figure S2**. All samples were collected using minimally invasive sampling techniques with sterile scalpels and then taken to the laboratory in an ice box for subsequent analyses. Six white spot samples (DTD2–DTD7) were subdivided where one was used for SEM while the other two were used for cultivation and biodiversity analyses. DTD1 was used only for microbial isolation and identification.

### Scanning Electron Microscopy

Minute samples from white spots were adhered to a conductive carbon tabs stuck on standard vacuum-clean stub and coated with gold. Gold coated samples were observed using a SEM (Hitachi S3600N). Images were obtained at magnifications between 600× and 2.5 k×, and at 20.0 kV for imaging.

### DNA Extraction and PCR Amplification

Total community genomic DNA was extracted from the samples using the MoBio PowerSoil^®^ DNA Isolation Kit (MO BIO Laboratories, Inc., Carlsbad, CA, United States) following the manufacturer’s protocol. Extracted DNA was diluted to 1 ng/μL using sterile water and then stored at -80°C for subsequent analyses.

Fungal ITS region sequencing followed previously described protocols (Kozich et al., 2013). Amplification of the fungal ITS1 gene region was performed using the ITS5-1737F/ITS2-2043R primers with barcodes attached that were unique to each sample (Supplementary Table S1). All PCR reactions were carried out using the Phusion^®^ High-Fidelity PCR Master Mix with GC Buffer (New England Biolabs, United Kingdom). Amplifications were carried out in a 30 μL mixture that included 15 μL of Master Mix (2X), a 0.5 μM final concentration of the forward and reverse primers, 10 ng of template DNA and nuclease-free water to 30 μL. PCR conditions consisted of an initial denaturation at 98°C for 1 min, followed by 30 cycles of 98°C for 10 s, 55°C for 30 s, and elongation at 72°C for 30 s, with a final extension at 72°C for 5 min.

To visualize PCR amplification success, an equal volume of 1X loading buffer (containing SYBR green) along with PCR products were loaded on a 2% agarose gel. Samples with amplicon bands in the range of 400–450 bp were chosen for further analyses. To pool samples, PCR products were mixed in equidensity ratios. Pooled PCR products were then purified using the Qiagen Gel Extraction Kit (Qiagen, Germany).

### High-Throughput Sequencing

The purified amplicons were prepared for Illumina sequencing by constructing a library using the TruSeq^®^ DNA PCR-Free Sample Preparation Kit (Illumina, United States) following the manufacturer’s recommendations. The final library concentrations and quality were checked using a Qubit@ 2.0 Fluorometer (Thermo Scientific) and an Agilent Bioanalyzer 2100 system, respectively. Lastly, 250 bp paired-end reads were generated for the library on a Hiseq2500 PE250 platform at the Novogene Bioinformatics Technology, Co., Ltd. (Beijing, China).

### Bioinformatic Analyses

Paired-end reads were assigned to samples based on their unique barcodes and then trimmed of barcode and primer sequences. Paired-end reads were merged using FLASH v.1.2.7 (Magoč and Salzberg, 2011) and the resultant sequences were considered as raw tags. Quality filtering of raw tags to obtain high-quality clean tags (Bokulich et al., 2013) was performed according to the QIIME v.1.7.0 (Caporaso et al., 2010) quality control protocol. Fungal tags were then compared against the UNITE reference database using the UCHIME algorithm (Edgar et al., 2011) to detect chimeric sequences, and sequences flagged as chimeras were then removed (Haas et al., 2011). The resultant high-quality sequences were used for further analyses. OTU clustering analysis was performed using Uparse v.7.0.1001 (Edgar, 2013). Sequences with ≥97% similarity in nucleotide identity were assigned to the same OTUs. Representative sequences for each OTU were then used for further annotation. Each representative fungal sequence was assigned a taxonomic classification using the BLAST algorithm and the UNITE Database (Kõljalg et al., 2013) in QIIME. Multiple sequence alignment was then conducted using the MUSCLE software package v.3.8.31 (Edgar, 2004). OTU abundances were then normalized across samples based on the sequence count in the sample with the least amount of sequences in order to limit bias from unequal sequencing depths. Subsequent analysis of beta diversity was performed based on the normalized abundance data. Beta diversity analysis was used to evaluate differences in community composition using the unweighted Unifrac distance metric as calculated with QIIME (v.1.7.0).

The raw sequencing data generated from this study has been deposited into the NCBI Sequence Read Archive (SRA) under the accession numbers SAMN06603620–SAMN06603625.

### Fungal Isolation and Identification

Fungal potato dextrose agar (PDA) medium was prepared for isolation and identification of fungi: 20% potatoes, 2% dextrose, 2% agar, and 1 L of tap water. These plates were incubated at 28°C for 5–30 days depending on the growth of fungi. Grown colonies were transferred to fresh plates containing PDA medium to obtain further pure isolates.

DNA extraction of pure fungal strains was performed using the CTAB method (Möller et al., 1992). Fungal 28S rRNA and ITS1-5.8S rRNA-ITS2 genes were amplified using the primers LR0R/LR7 and ITS1/ITS4, respectively (White et al., 1990; Kruys et al., 2015). PCR reaction mixtures consisted of a total volume of 50 μL containing 2 μL of genomic DNA, 5 μL of 10× Reaction Buffer, 4 μL of 2.5 mM dNTP mix, 2 μL of 10 μM forward primer, 2 μL of 10 μM reverse primer, 0.5 μL of 5 U/μL Transtaq-T DNA polymerase (TransGen Biotech, China), and ddH_2_O to 50 μL. The PCR conditions were 95°C for 3 min, followed by 32 cycles of 30 s at 95°C, 30 s at 55°C for ITS region amplification, and 20 s at 72°C, with a final extension of 5 min at 72°C. Parameters for amplifying the 28S rRNA genes were identical except extension was conducted at 72°C for 1 min. PCR products were detected by electrophoresis in 1% agarose gels and purified using a AxyPrep PCR Clean Up Kit (Axygen, United States).

The purified PCR products were sequenced by GENEWIZ (Beijing, China) and sequence identities were analyzed using the National Center for Biotechnology Information (NCBI) BLASTn program^1^ and the GenBank database. Cladograms showing evolutionary relationships were conducted using the Molecular Evolutionary Genetics Analysis software package (MEGA, v.5.05) using the neighbor-joining method. Confidence in tree topology was estimated with 1,000 bootstrap replicates.

### Enzymatic Characteristics of Dominant Fungi

To test ligninolytic enzyme and cellulase activity, two different media were prepared: (i) fungal PDA plates containing 0.04% (v/v) guaiacol (ii) CMC agar medium consisting of 0.2% NaNO_3_, 0.1% K_2_HPO_4_, 0.05% MgSO_4_, 0.05% KCl, 0.2% carboxymethylcellulose (CMC) sodium salt, 0.02% peptone, 1.7% agar, and 1 L of tap water. Gram’s iodine consisted of 2.0 g KI and 1.0 g iodine in 300 ml distilled water which was used to flood CMC plates for 3–5 min. All media were autoclaved for 20 min at 121°C.

Strain WY-DT1 was cultivated on PDA-guaiacol and CMC plates at 14°C for 7 days. Strain NK-DT1 was cultivated on PDA-guaiacol and CMC plates at 28°C for 4 days.

## Results

### Microscopic Observation

To investigate microbial deterioration of tomb M2, representative samples of the white spots that were present on the surface of the *ticou* wall were observed by SEM. An abundance of mycelia was identified and fungal hyphae with a diameter of 2–3 μm were clearly discerned (**Figures 2B,C** and **Supplementary Figure S2**). The micrographs showed numerous spores on the surface of the sample, which could not be attributed to a genus due to their fragmentary state. The abundance of mycelium and spores indicated that significant fungal colonization of the tomb had occurred.

### Fungal Community Analysis

**FIGURE 3:**
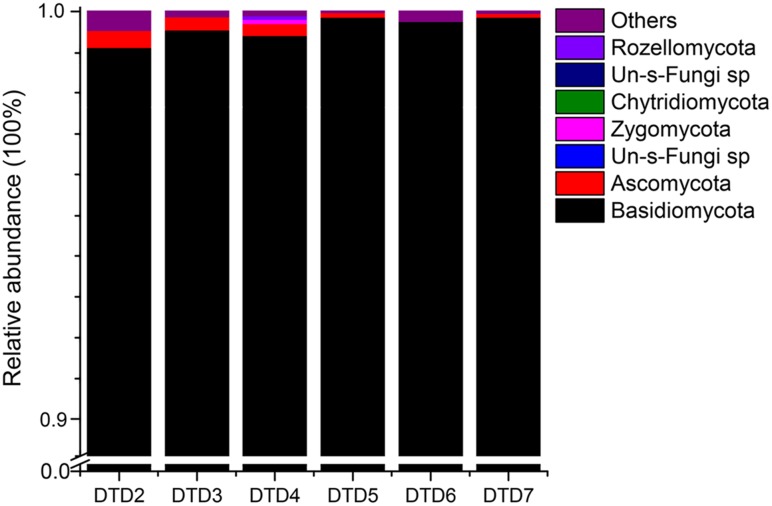
Relative abundances of the fungal phyla among samples. Relative abundances (out of 100%) for each sample are shown based on ITS sequencing. Fungal phyla are colored according to the legend on the right.

High-throughput sequencing was carried out on an Illumina Hiseq2500 PE250 platform to assess the diversity and variability of the fungal communities colonizing the wooden tomb. A total of 1,457,734 fungal reads were recovered after filtering low-quality reads and chimeras. The distribution of identified and unidentified fungal phyla among the six samples is summarized in **Figure 3**. In total, five fungal phyla were detected in the six samples. Basidiomycota were the most dominant phylum in all six samples, accounting for 99.10, 99.52, 99.38, 99.71, 99.59, and 99.75% of the fungal communities, with an average relative abundance of 99.50%. Ascomycota were also present in the six samples and comprised 0.44, 0.33, 0.37, 0.23, 0.04, and 0.19% of the fungal communities with an average relative abundance of 0.27%. The other three phyla that were present, Zygomycota, Chytridiomycota, and Rozellomycota accounted for the remaining 0.23% of the total abundances. In total, there were 114 fungal genera that were detected among the samples (Supplementary Table S2), and the dominant genera were similar in all samples (**Table 1**). Among the 10 most abundant fungal genera, *Hypochnicium*, *Cortinarius*, and *Geminibasidium* were present in all samples and *Hypochnicium* was the most abundant genus across all samples, accounting for 98.61–98.45% of the community totals, with an average abundance of 99.22%. Other fungal genera only comprised the minute remainder.

**Table 1:** Genus-level relative abundances and community composition of samples DTD2–7.

Genus	DTD2 (%)	DTD3 (%)	DTD4 (%)	DTD5 (%)	DTD6 (%)	DTD7 (%)
*Hypochnicium*	98.61	99.41	99.19	99.36	99.30	99.45
*Ramariopsis*	0	<0.01	0	<0.01	0.28	0
*Cortinarius*	0.21	0.01	0.02	0.13	<0.01	0.14
*Geminibasidium*	0.14	<0.01	0.01	0.14	<0.01	0.09
*Eupenicillium*	0.01	<0.01	0.10	<0.01	0	<0.01
*Oidiodendron*	0.06	0.01	0.01	0.01	0	<0.01
*Trechispora*	0.05	0.01	0.03	<0.01	0	<0.01
*Hygrophorus*	<0.01	0.01	0.04	0	0	0.01


### Cultivation of the Dominant Fungal Populations

A filamentous fungal isolate, ‘WY-DT1,’ was isolated from the white spot samples. The isolate grew slowly and the color of the colonies were white to cream and hypochnoid, while hyphae diameter was ∼2–3 μm (**Supplementary Figure S3**). Analysis of the isolate’s ITS sequence indicated that it displayed 99% sequence similarity with *Hypochnicium* spp. and was thus identified as *Hypochnicium* sp. WY-DT1 (KP980549, Supplementary Table S3). Phylogenetic analyses indicated that strain WY-DT1 belongs to the genus *Hypochnicium* and belongs to a distinct subclade along with *Hypochnicium bombycinum* (FN552537) and *Hypochnicium lyndoniae* (JX124704).

To confirm that the isolated strain *Hypochnicium* sp. WY-DT1 was the fungus responsible for wood deterioration within the tomb, strain WY-DT1 was inoculated onto PDA-guaiacol plates. Lignocellulolytic enzymes catalyze the oxidative polymerization of guaiacol to form reddish brown zones in the medium and wood degrading ability is directly proportional to the size and depth of the reddish brown zones (Viswanath et al., 2008). That is, the larger and deeper the reddish brown zones, the stronger the wood degrading ability. Crimson circles indicate that strain *Hypochnicium* sp. WY-DT1 can significantly degrade lignin at low temperatures. To further test whether the strain can degrade cellulose, it was inoculated on to carboxymethylcellulose (CMC) plates (**Supplementary Figure S3**). The presence of clear and distinct zones indicated that the strain has the ability to metabolize cellulose. Taken together, the cultivation results indicated that strain WY-DT1 is capable of wood biodeterioration and may be the fungus responsible for tomb deterioration.

Another fungal strain, NK-DT1, was isolated from the sample DTD1 (**Supplementary Figure S4**). Cultures were fast growing, white to grayish-white, downy, and often exhibited a broadly zonate or lobed surface appearance on PDA plates. NK-DT1 displayed 99% ITS sequence similarity to *Mortierella* spp. (Supplementary Table S3). Morphological and molecular biological results led to the determination that the strain belonged to the *Mortierella* genus and was thus designated *Mortierella* sp. NK-DT1 (KY779731). The *Mortierella* genus was detected in five samples (excluding DTD6) and also possessed high cellulolytic activity (Supplementary Table S2 and **Figure S4**).

## Discussion

Scanning electron microscopy revealed clear evidence for the association of filamentous fungi with the white spots. The high abundance of spores that were identified by SEM could indicate how the white spots were able to spread so quickly throughout the wooden tomb. The vast numbers of spores that were present could be an indication of the fungi’s harmful contribution to the tomb’s rapid deterioration.

High-throughput sequencing on the Illumina Hiseq2500 platform revealed 114 fungal genera that belonged to five phyla. The use of high-throughput sequencing can provide deeper assessments of the microbial communities that are related to the biodeterioration of cultural heritage items. For instance, using less-throughput techniques (i.e., clone-library based sequencing), only 14 fungal genera were identified in a study of wooden stairs (Piñar et al., 2016). The Basidiomycota phylum accounted for nearly all of the reads in each sample analyzed here (99.10–99.75% relative abundances of the communities), with an average relative abundance of 99.50%. Basidiomycota, Ascomycota, Zygomycota, Chytridiomycota, and Rozellomycota are often responsible for the biodeterioration of wooden relics (Irbe, 2010; Irbe et al., 2012; Palla and Barresi, 2017).

Although a number of fungal genera were detected in the samples from the tomb, the *Hypochnicium* genus was dominant in all samples, and accounted for more than 98% of the total fungal communities. *Hypochnicium* belongs to the Basidiomycota phylum and the reproductive hyphae exhibit clamp connections that are colorless and thin or exhibit thick walls. The hyphae have a mixed arrangement and produce branches at the clamp connections. There are a total of 33 currently accepted *Hypochnicium* species, with 12 species of *Hypochnicium* having been identified in China alone (Qin and He, 2013). The genus is one of the corticoid fungi which have proved to be responsible for active wood decay in buildings (Irbe et al., 2012). *Hypochnicium* can also exist in extreme polar environments such as Antarctica, which indicates that some members of the genus may have lower growth temperature requirements (Held and Blanchette, 2017). Of relevance to cultural heritage studies, *Hypochnicium* sp. is one of deteriorative agents of the Latvian Ethnographic Open Air Museum (Irbe et al., 2012).

That *Hypochnicium* can exhibit the ability to grow at low temperatures may explain why *Hypochnicium* comprised a large proportion of the fungal communities in the tomb which features temperatures between 10 and 16°C. Our cultivation analyses indicate that *Hypochnicium* sp. WY-DT1 possesses lignolytic and cellulolytic enzymes that could be responsible for the digestion of complex organic components of wood including cellulose, hemicelluloses, and lignin. It follows that the wooden structure of the tomb M2 could feasibly provide nutrients for the optimal growth and wood-degrading activity of the *Hypochnicium* sp. WY-DT1 isolate that was identified here.

Another isolate that we recovered, *Mortierella* sp. NK-DT1, belongs to the Zygomycota phylum. *Mortierella* spp. typically live as saprotrophs in soils, growing on decaying leaves and other organic material and they also possess the ability to degrade cellulose (Štursová et al., 2012). Our results indicated that *Mortierella* sp. NK-DT1 exhibited high cellulase activity. Accordingly, the fungal strain is likely to also contribute damage to the wooden structure of the tomb and must be regarded as a threat along with the *Hypochnicium* sp. WY-DT1 that was identified. In addition to the *Hypochnicium* and *Mortierella* species that were identified, other genera that comprised minor components of the fungal communities (e.g., as *Penicillium* and *Aspergillus*), and exhibit cellulolytic activities, were also present and could be potential deteriorative threats for the wooden tomb.

Fungi in the genera *Antrodia*, *Athelia*, *Gloeophyllum*, *Hyphoderma*, *Hyphodontia*, *Botryobasidium*, and *Postia* have all been associated with wood in archeological heritage materials. The majority of fungi in wooden structures were corticoid species which they caused a typical white rot. The biodeterioration potential of these genera has been described and discussed in context of their respective threatened materials (Irbe et al., 2012). Wood from the tomb of King Midas (700 B.C., Turkey) showed soft rot decay. The extreme environmental conditions of the tomb such as low moisture and high pH inhibited white and brown rot fungi but favored soft rot. Wood from Egyptian tombs (3000–1000 B.C.) displayed degradation caused by brown rot fungi and in a few cases by soft rot fungi (Blanchette et al., 1991). Unlike these previous cases, we found a significant proportion of white rot fungi which in the case was *Hypochnicium* in the tomb. The distinctiveness of this genera’s dominance in the M2 tomb suggests that the environment of the tomb (high moisture, low temperature) has led to the specific colonization and potential adaptation of these species to the tomb environment. Further analyses could indicate which adaptations have led to the prevalence of this species in this particular environment.

Cellulose and hemicellulose are normally surrounded by lignin within wood which acts as a barrier between carbohydrates and cellulolytic organisms. Fungi, including white, brown, and soft rot types, are generally known to decompose lignin and hemicellulose in lignocellulosic biomass (Sánchez, 2009). Among the white rot fungi, more than 1,500 species have the ability to degrade lignin (Tian et al., 2012). These fungi are even applied in the biological pre-treatment of biomass in the process of biofuel production (Itoh et al., 2003). In addition to biological pre-treatment, they can also be used in other bioconversion processes including wastewater treatment, bio-pulping, and the bioconversion of forest and agricultural wastes to animal feeds (Nilsson et al., 2006; Levin et al., 2007; van Kuijk et al., 2015). In the context of the tomb, the lignolytic activity of these isolates may be the main causative mechanism underpinning biodegradation, despite their beneficial application in many industrial processes.

Protective measures should be applied to control the current biodeterioration of the M2 tomb and aid conservation efforts. The application of biocides is one of the most important means to control microbial deterioration (Polo et al., 2010; Ríos et al., 2012; Diaz-Herraiz et al., 2013; Coutinho et al., 2016). However, the application of biocides can exert a selective pressure on microbial populations, and the populations may then develop biocide-resistance mechanisms, or the affected populations may be replaced by others that could be even more harmful to the artifacts (Mitchell and McNamara, 2010). One such example is in the French Lascaux Caves, where a series of biocide treatments were applied that triggered new microbial outbreaks (Martin-Sanchez et al., 2012). Thus, the application of biocides in the tomb is currently a less optimal approach and not recommended at present. Therefore, frequent cleaning and other mechanical removal procedures should be the current top priority to mitigate fungal biodeterioration.

Lastly, humidity is one of the most important factors to consider when preserving artifacts. Optimal humidity levels for white-rot and brown-rot fungi are in the range of 40–80% (Blanchette, 2000). Sterile, deionized water has been sprayed throughout the M2 tomb to maintain continuously waterlogged conditions. During this process, our observations have indicated that the white spots grew faster when spraying ceased. Thus, maintaining waterlogged conditions may be an important strategy in order to control the contamination of the tomb by the white spot fungal communities.

## Conclusion

Our study represents the first high-throughput community sequencing and cultivation analyses of fungi associated with an ancient wooden tomb that exhibited obvious biodeterioration. SEM revealed clear evidence of filamentous fungi associated with the white spots. Although five fungal phyla including Basidiomycota, Ascomycota, Zygomycota, Chytridiomycota, and Rozellomycota were identified, Basidiomycota represented by *Hypochnicium* genus dominated the fungal communities with an average abundance of 99.22% in six samples. For the isolates obtained, we found the two species, including *Hypochnicium* sp. WY-DT1 and *Mortierella* sp. NK-DT1, possess the ability to degrade cellulose or lignin. Finally, in addition to controlling the environment as a means to inhibit microorganism deterioration activity, efficient monitoring and protective measures should be applied to mitigate current fungal biodeterioration. Although the investigation provided a basic understanding on the dominant members of the fungal community that are likely to be responsible for contamination and potential deterioration of the M2 tomb, the reason behind the *Hypochnicium* contamination is yet unclear. Additional research is required in order to better understand the adaptations and ecological traits that allowed the genus to dominate the M2 tomb-contaminating communities.

## Author Contributions

All authors contributed to this work. JP conceived and planned the research. ZL performed statistical analyses and wrote the manuscript. YW and QL extracted DNA, cultured and tested the isolates. XP and QG organized the sampling trips and provided access to the sampling sites. TF and CH assessed lignocellulose activity. QM and XZ edited the manuscript.

## Conflict of Interest Statement

The authors declare that the research was conducted in the absence of any commercial or financial relationships that could be construed as a potential conflict of interest.

## Footnotes

1 https://blast.ncbi.nlm.nih.gov/Blast.cgi

## Supplementary Material

FIGURE S1 The structure of the tomb M2. **(A)** Cross-section drawing of the tomb. **(B)** The plan of the tomb. S1–S12 are 12 outer storage chambers. HB, HN, QB, QN, ZB1, ZB2, ZN1, and ZN2 are eight side chambers. WD, SD, and ND refer to west doorway, south doorway and north doorway, respectively. FC, front chamber; CC, central chamber; RC, rear chamber. Red stars indicate the sampling positions.

FIGURE S2 White spots on the *ticou* wall of the tomb. **(A)** The samples with the white spots (DTD3–DTD7) were collected for cultivation analyses and high-throughput sequencing in March 2015. **(B,C)** Scanning electron micrograph of white spots.

FIGURE S3 Colony morphological appearances of strain *Hypochnicium* sp. WY-DT1 on different media. Colony **(A)** and micro-morphology features **(B)** of the culprit fungus at 100× magnification. The white bar represents 10 μm. **(C)** Neighbor-joining phylogenetic tree based on the ITS sequence (approximately 640 bp) of strain WY-DT1, including representatives of the most closely related strains and additional members of the genus *Hypochnicium*. Bootstrap values are given at the nodes as a percentage of 1,000 bootstrap replicates. **(D)** The culprit deteriorative fungus was cultivated on PDA-guaiacol plates. The crimson circle indicated that the fungus can degrade lignin significantly at low temperatures. **(E)** Effect of Gram’s iodine flooding on cellulolytic zone in CMC plates. The transparent circle indicates that the fungus can produce cellulose.

FIGURE S4 Colony morphological appearances of strain *Mortierella* sp. NK-DT1 on different media. **(A,B)** Colony features of NK-DT1. **(C)** Strain NK-DT1 was cultivated on PDA-guaiacol plates. Results showed the fungus have no ability to degrade lignin. **(D)** The transparent circle indicates that NK-DT1 can also produce cellulose.
