# Supplementary figures and images for: Identification of Fungal Communities Associated with the Biodeterioration of Waterlogged Archeological Wood in a Han Dynasty Tomb in China
Source: Front Microbiol. 2017 Aug 24;8:1633. doi: 10.3389/fmicb.2017.01633 (PMC5575450; doi:10.3389/fmicb.2017.01633)

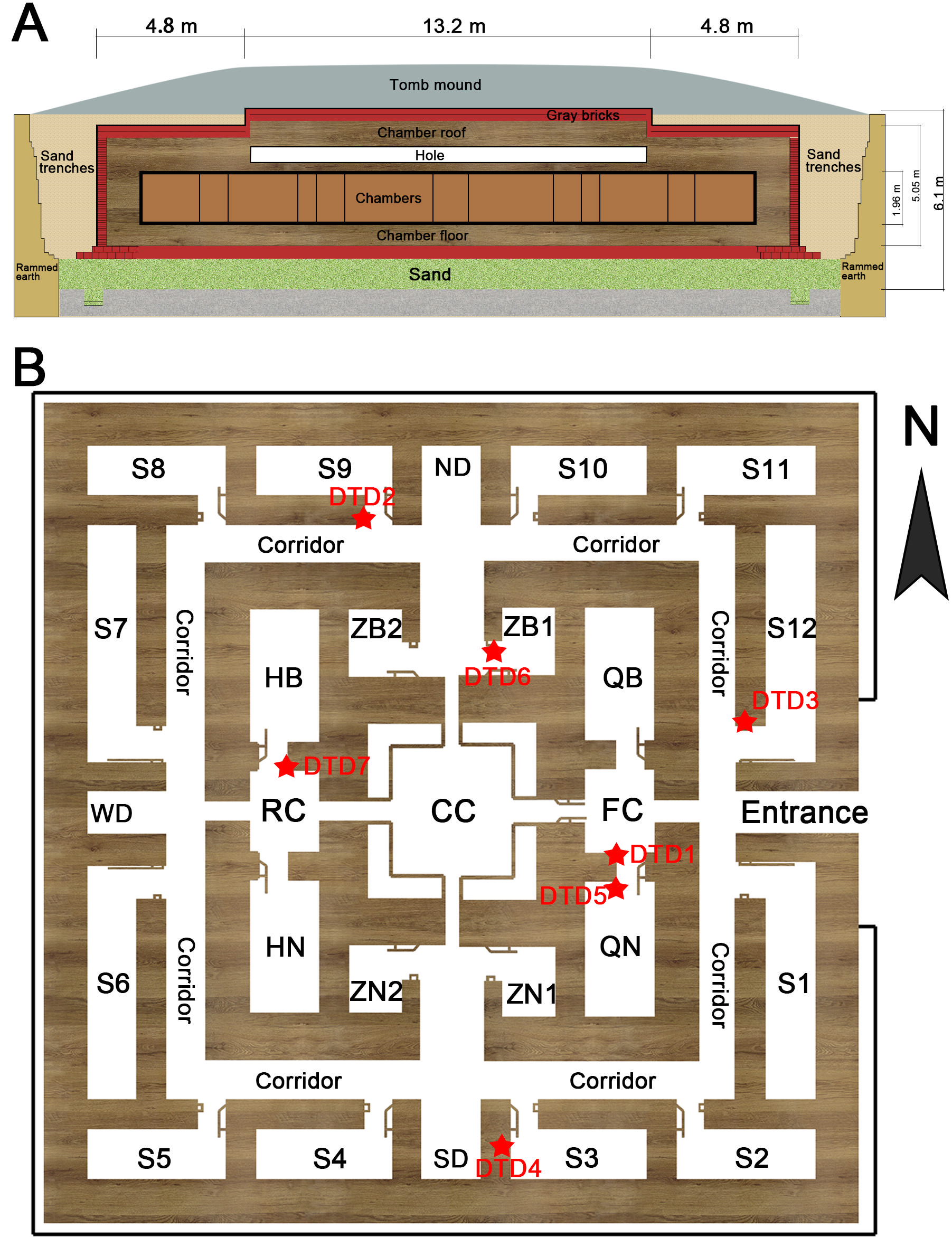

Supplement: FIGURE S1 — The structure of the tomb M2. (A) Cross-section drawing of the tomb. (B) The plan of the tomb. S1–S12 are 12 outer storage chambers. HB, HN, QB, QN, ZB1, ZB2, ZN1, and ZN2 are eight side chambers. WD, SD, and ND refer to west doorway, south doorway and north doorway, respectively. FC, front chamber; CC, central chamber; RC, rear chamber. Red stars indicate the sampling positions. [file Image_1.TIF]

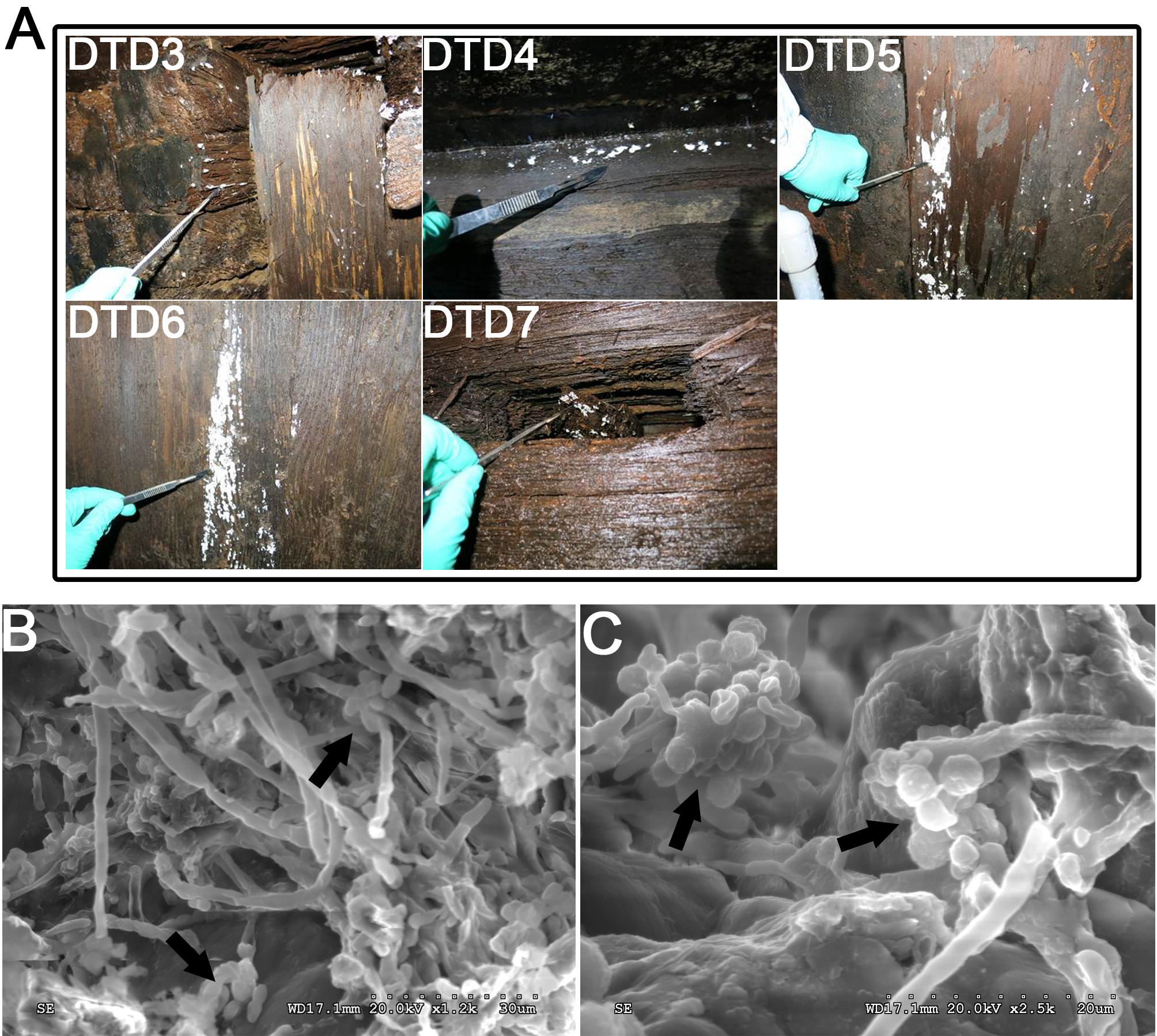

Supplement: FIGURE S2 — White spots on the ticou wall of the tomb. (A) The samples with the white spots (DTD3–DTD7) were collected for cultivation analyses and high-throughput sequencing in March 2015. (B,C) Scanning electron micrograph of white spots. [file Image_2.TIF]

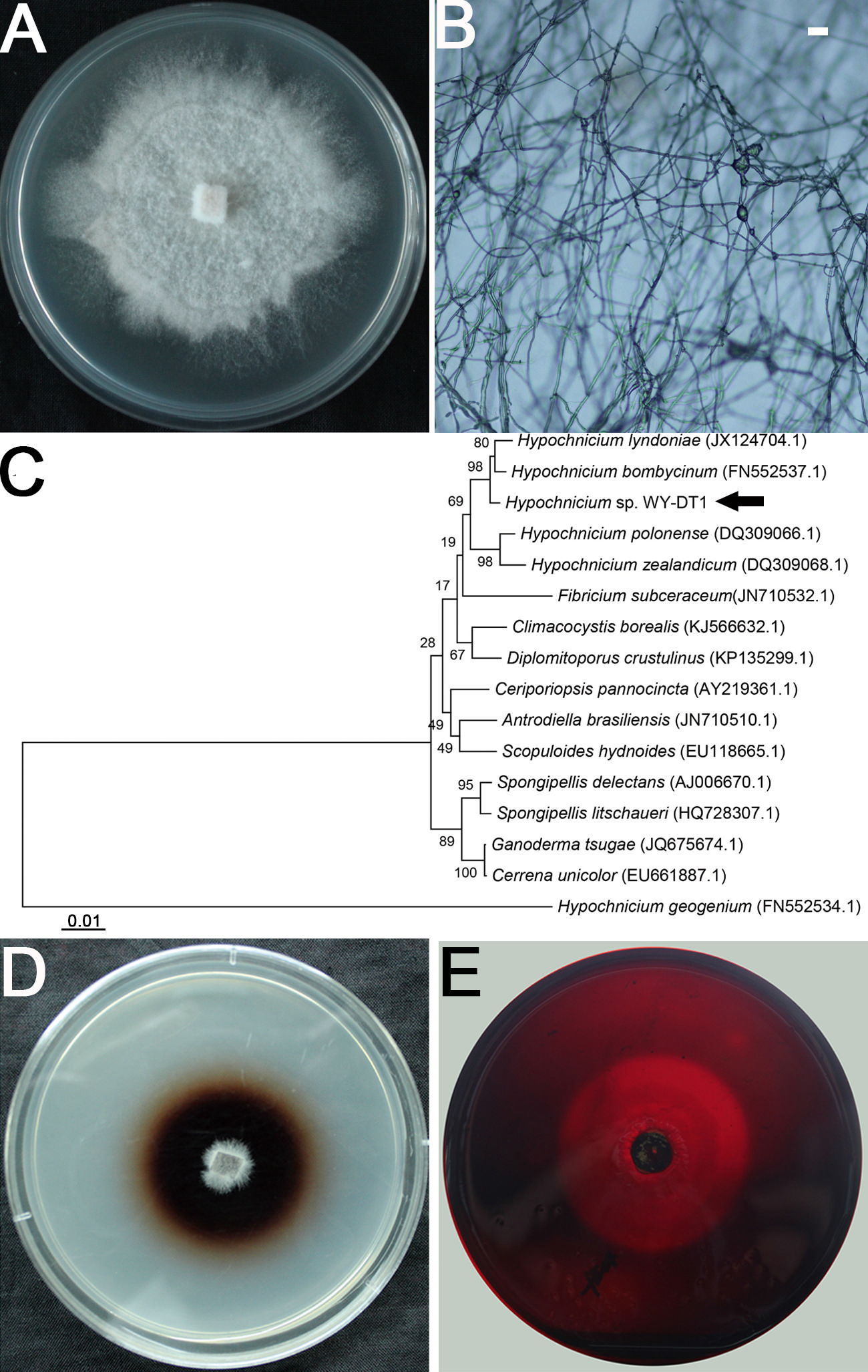

Supplement: FIGURE S3 — Colony morphological appearances of strain Hypochnicium sp. WY-DT1 on different media. Colony (A) and micro-morphology features (B) of the culprit fungus at 100× magnification. The white bar represents 10 μm. (C) Neighbor-joining phylogenetic tree based on the ITS sequence (approximately 640 bp) of strain WY-DT1, including representatives of the most closely related strains and additional members of the genus Hypochnicium. Bootstrap values are given at the nodes as a percentage of 1,000 bootstrap replicates. (D) The culprit deteriorative fungus was cultivated on PDA-guaiacol plates. The crimson circle indicated that the fungus can degrade lignin significantly at low temperatures. (E) Effect of Gram’s iodine flooding on cellulolytic zone in CMC plates. The transparent circle indicates that the fungus can produce cellulose. [file Image_3.TIF]

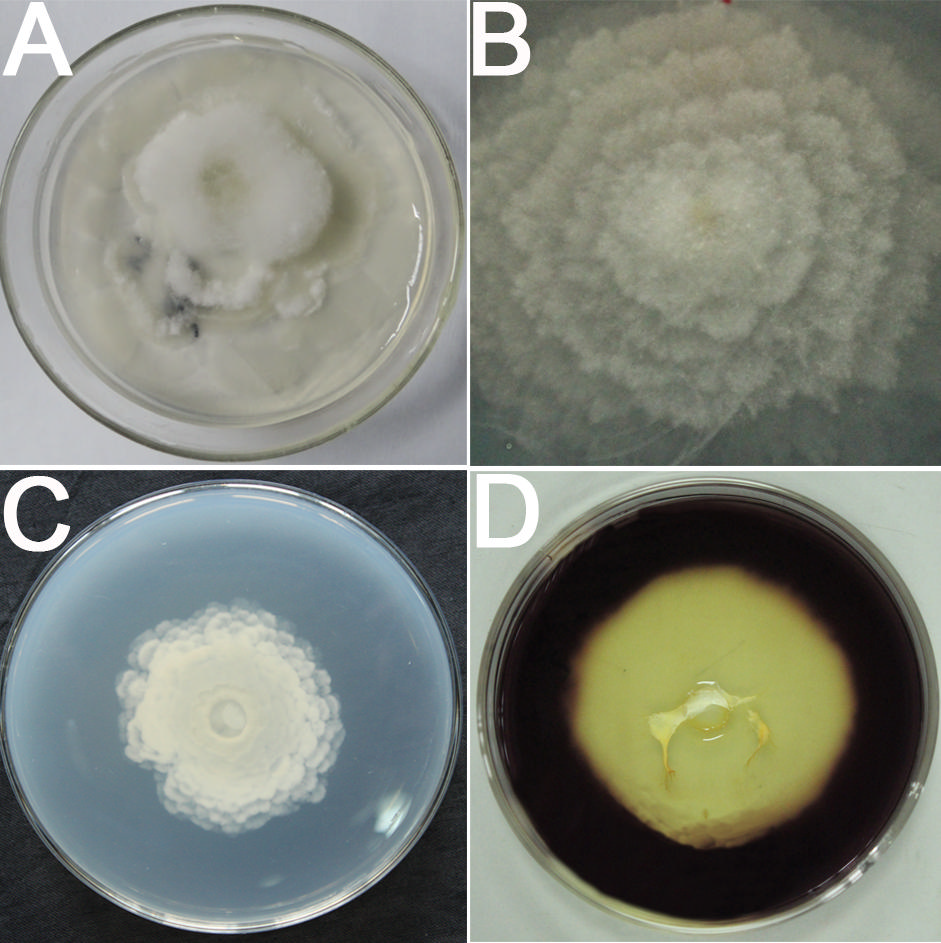

Supplement: FIGURE S4 — Colony morphological appearances of strain Mortierella sp. NK-DT1 on different media. (A,B) Colony features of NK-DT1. (C) Strain NK-DT1 was cultivated on PDA-guaiacol plates. Results showed the fungus have no ability to degrade lignin. (D) The transparent circle indicates that NK-DT1 can also produce cellulose. [file Image_4.TIF]
