# Supplementary material for: Identification of Fungal Communities Associated with the Biodeterioration of Waterlogged Archeological Wood in a Han Dynasty Tomb in China
Source: Front Microbiol. 2017 Aug 24;8:1633. doi: 10.3389/fmicb.2017.01633 (PMC5575450; doi:10.3389/fmicb.2017.01633)
Supplement: Supplementary file 5 [file Data_Sheet_1.PDF]

Supplementary Material

Identification of fungal communities associated with the biodeterioration of waterlogged archaeological wood in a Han Dynasty tomb in China

Zijun Liu[1#], Yu Wang[1#], Xiaoxuan Pan[2], Qinya Ge[2], Qinglin Ma[2], Qiang Li[3], Tongtong Fu[1], Cuiting Hu[1], Xudong Zhu[1] and Jiao Pan[1*]

[1] Key Laboratory of Molecular Microbiology and Technology, Ministry of Education, College of Life Sciences, Nankai University, Tianjin, China

[2] Chinese Academy of Cultural Heritage, Beijing, China

[3] Laboratory of Cultural Relics Conservation Materials, Department of Chemistry, Zhejiang University, Hangzhou, China

[#] **These authors contributed equally to this work.**

***Correspondence:**

Dr. Jiao Pan

Email: panjiaonk@nankai.edu.cn

Table S1. Primer sequences used in this study.

Primer pair	Targeting region	Sequence (5`-3`)
ITS5-1737F/ ITS2-2043R	ITS 1	Forward: GGAAGTAAAAGTCGTAACAAGG<br>Reverse: GCTGCGTTCTTCATCGATGC
LR0R/LR7	28S rRNA	Forward: ACCCGCTGAACTTAAGC<br>Reverse: TACTACCACCAAGATCT
ITS1/ITS4	ITS1-5.8S rRNA-ITS2	Forward: TCCGTAGGTGAACCTGCGG<br>Reverse: CCTCCGCTTATTGATATGC

Table S2. The distribution of the identified and unidentified fungal genera in the six samples.

Taxonomy	DTD2	DTD3	DTD4	DTD5	DTD6	DTD7
Hypochnicium	0.986097	0.994073	0.991855	0.993588	0.992965	0.994507
Ramariopsis	0	1.87E-05	0	3.47E-06	0.002808	0
Cortinarius	0.002093	0.000131	0.00023	0.001281	4.69E-05	0.001433
Un--s-Helotiales sp	0.001625	0.000994	0.000759	0.001229	0	0.000221
Geminibasidium	0.001417	1.87E-05	0.000127	0.001392	1.17E-05	0.00092
Eupenicillium	0.000113	6.07E-05	0.001001	4.17E-05	0	8.45E-05
Un--s-fungal sp	0	4.67E-06	0	0	0.000885	0
Oidiodendron	0.000609	0.000103	0.000189	0.000135	0	3.25E-05
Un--s-Pseudeurotiaceae sp	0.00052	4.20E-05	4.10E-05	5.90E-05	0	0.000143
Trechispora	0.000515	0.000112	0.000332	5.21E-05	0	9.75E-06
Hygrophorus	8.98E-05	0.000121	0.000443	0	0	0.00014
Un--s-Trichocomaceae sp	9.45E-06	0	0.000394	0	2.35E-05	3.25E-05
Un--s-Herpotrichiellaceae	2.83E-05	0.000378	8.21E-06	1.04E-05	0	7.80E-05
Un--s-Leotiomycetes sp	6.14E-05	0.000359	5.33E-05	1.39E-05	5.86E-06	4.23E-05
Umbelopsis	0.000104	3.27E-05	0.000283	0.000271	0	0.000341
Jattaea	0.000331	4.67E-06	0	0	0	3.25E-06
Un--s-Agaricales sp	0.00018	0.000173	0.000283	0.000264	1.17E-05	9.75E-06
Un--s-Ascomycota sp	0.000241	0.000121	0.000181	0.000163	0	0.000224
Oidiodendron	0.000132	9.33E-06	0.000127	0.000215	0	0.000124
Phialocephala	8.03E-05	0.000196	0.000144	0.000194	0	7.80E-05
Un--s-Helotiaceae sp	1.42E-05	0.000196	0.000168	6.94E-06	0	0
Cryptococcus	6.14E-05	0.000177	3.28E-05	5.55E-05	0	0.00013
Hypomyces	1.89E-05	1.87E-05	0	0	0.000164	3.25E-06
Un--s-Dothideomycetes sp	4.25E-05	0.000163	5.33E-05	0	0	3.25E-06
Cistella	1.42E-05	0	4.10E-06	2.43E-05	0	0.000146
Cladophialophora	8.50E-05	7.47E-05	8.21E-05	3.47E-06	0	0.000143
Pseudogymnoascus	1.42E-05	8.87E-05	2.87E-05	6.94E-06	0	0.000133

(*Continued*)

Table S2. (***Continued***)

Taxonomy	DTD2	DTD3	DTD4	DTD5	DTD6	DTD7
Piloderma	0	0	0.000131	0	0	3.25E-05
Un--s-Hypocreaceae sp	4.72E-06	1.40E-05	0	6.94E-06	0	0.00013
Un--s-Sebacinales Group B	0	0.000126	0	7.64E-05	0	3.25E-06
Un--s-Fungi sp	0.000123	6.53E-05	4.10E-06	0	0	2.60E-05
Chytriomyces	0	0.000112	0	3.47E-06	0	0
Un--s-Agaricomycetes sp	0	0	0	0	0	0.000107
Un--s-Chaetothyriales sp	0	0.000103	0	6.94E-06	0	1.95E-05
Russula	8.03E-05	1.87E-05	9.44E-05	9.72E-05	0	3.25E-06
Pseudotomentella	8.98E-05	9.33E-06	4.10E-06	1.04E-05	5.86E-06	3.25E-06
Un--s-Microbotryomycetes	8.50E-05	4.67E-06	0	0	0	3.25E-06
Penicillium	4.72E-06	4.67E-06	2.87E-05	3.47E-06	8.21E-05	1.30E-05
Mortierella	5.20E-05	2.94E-04	2.87E-05	1.39E-05	0	1.37E-04
Umbelopsis	3.78E-05	0	7.39E-05	1.74E-05	0	6.50E-06
Un--s-Lecanoromycetes sp	4.72E-06	9.33E-06	6.98E-05	3.12E-05	0	2.28E-05
Arthrobotrys	0	0	1.64E-05	0	5.86E-05	6.50E-06
Verticillium	2.83E-05	4.67E-06	5.33E-05	0	0	0
Aspergillus	9.45E-06	0	4.10E-06	0	5.28E-05	0
Un--s-Thelephoraceae sp	4.72E-06	9.33E-06	3.69E-05	5.21E-05	1.17E-05	6.50E-06
Oidiodendron	5.20E-05	0	0	3.47E-06	0	3.58E-05
Un--s-Trechisporales sp	9.45E-06	5.13E-05	4.51E-05	2.08E-05	1.76E-05	2.28E-05
Un--s-Hyaloscyphaceae sp	4.72E-06	5.13E-05	0	0	0	9.75E-06
Un--s-Sporidiobolales sp	0	0	4.10E-05	4.86E-05	0	9.75E-06
Rickenella	4.72E-05	0	1.23E-05	0	0	0
Leptodontidium	9.45E-06	4.67E-05	2.05E-05	6.94E-06	0	6.50E-06
Lecanicillium	4.25E-05	1.40E-05	1.23E-05	3.47E-06	0	3.25E-06
Ceratobasidium	0	4.20E-05	4.10E-06	3.47E-06	0	0
Cryptococcus	3.78E-05	0	0	1.04E-05	0	1.30E-05

(*Continued*)

Table S2. (*Continued*)

Taxonomy	DTD2	DTD3	DTD4	DTD5	DTD6	DTD7
Mucor	0	0	3.69E-05	0	0	9.75E-06
Inocybe	0	0	0	0	0	3.58E-05
Un--s-Ceratobasidiaceae sp	1.89E-05	4.67E-06	3.28E-05	2.43E-05	0	3.25E-06
Sistotrema	0	0	0	3.12E-05	0	1.63E-05
Meliniomyces	0	9.33E-06	2.87E-05	0	0	0
Un--s-Eurotiales sp	2.83E-05	0	0	0	0	0
Leuconeurospora	2.83E-05	0	0	0	0	0
Doratomyces	0	0	2.46E-05	0	0	0
Suillus	2.36E-05	9.33E-06	2.46E-05	1.04E-05	0	6.50E-06
Arachnopeziza	1.89E-05	9.33E-06	2.46E-05	0	0	0
Un--s-Ascomycota sp	2.36E-05	0	0	0	0	0
Pochonia	2.36E-05	0	0	3.47E-06	0	3.25E-06
Mrakia	0	2.33E-05	0	0	0	0
Scleroderma	0	2.33E-05	0	0	1.17E-05	0
Lachnum	0	2.33E-05	0	0	0	0
Geomyces	0	0	0	6.94E-06	0	2.28E-05
Cylindrosympodium	1.42E-05	4.67E-06	2.05E-05	6.94E-06	0	0
Boletinus	0	0	0	0	0	1.95E-05
Un-s-Rozellomycota sp	1.89E-05	9.33E-06	4.10E-06	0	0	0
Phaeoisaria	0	0	0	0	1.76E-05	0
Oidiodendron	4.72E-06	4.67E-06	8.21E-06	3.47E-06	0	1.63E-05
Tomentella	1.42E-05	4.67E-06	0	3.47E-06	1.17E-05	1.30E-05
Scedosporium	1.42E-05	0	4.10E-06	0	0	0
Chaetosphaeria	1.42E-05	0	8.21E-06	1.04E-05	0	0
Geastrumia	0	1.40E-05	4.10E-06	6.94E-06	0	3.25E-06
Venturia	0	1.40E-05	0	0	0	3.25E-06
Hypholoma	0	1.40E-05	0	0	0	0

(*Continued*)

Table S2. (*Continued*)

Taxonomy	DTD2	DTD3	DTD4	DTD5	DTD6	DTD7
Rhizoscyphus	0	1.40E-05	8.21E-06	0	0	0
Un-s-Chaetosphaeriaceae sp	0	9.33E-06	0	1.39E-05	0	9.75E-06
Hypocrea	4.72E-06	0	0	1.04E-05	0	1.30E-05
Un-s-Hyaloscyphaceae sp I	0	4.67E-06	1.23E-05	0	0	3.25E-06
Hohenbuehelia	4.72E-06	0	1.23E-05	3.47E-06	0	3.25E-06
Un-s-Sebacinaceae sp	0	0	1.23E-05	0	0	0
Trichoderma	0	0	1.23E-05	0	5.86E-06	0
Ilyonectria	0	4.67E-06	4.10E-06	0	5.86E-06	9.75E-06
Eleutheromyces	9.45E-06	0	0	0	0	0
Rotiferophthora	9.45E-06	0	0	3.47E-06	0	0
Xenopolyscytalum	9.45E-06	0	0	0	0	6.50E-06
Cystodendron	0	9.33E-06	0	0	0	0
Un-s-Boletales sp	4.72E-06	9.33E-06	0	0	0	0
Leptosphaeria	0	0	8.21E-06	0	0	0
Oidiodendron	4.72E-06	0	8.21E-06	3.47E-06	5.86E-06	3.25E-06
Ombrophila	0	0	8.21E-06	0	0	0
Un-s-Albatrellaceae sp	0	0	8.21E-06	0	0	0
Lactarius	0	0	0	6.94E-06	0	0
Un-s-Dermateaceae sp	0	0	0	6.94E-06	0	0
Hygrocybe	0	0	0	0	0	6.50E-06
Rasamsonia	0	0	0	0	0	6.50E-06
Plectania	0	0	0	0	0	6.50E-06
Cladosporium	4.72E-06	0	0	0	5.86E-06	0
Laetisaria	0	0	4.10E-06	3.47E-06	5.86E-06	3.25E-06
Acremonium	4.72E-06	0	4.10E-06	0	0	0
Didymella	0	4.67E-06	0	3.47E-06	0	0
Mucor	0	4.67E-06	0	0	0	3.25E-06

(*Continued*)

Table S2. (*Continued*)

Taxonomy	DTD2	DTD3	DTD4	DTD5	DTD6	DTD7
Cryptosporiopsis	0	3.73E-05	0	0	0	0
Minimedusa	0	0	4.10E-06	0	0	3.25E-06
Others	0.004474	0.001083	0.002171	0.00041	0.002779	0.000166

Table S3. Gene-based identification results of isolates.

Strain name	Sequencing region	Closest match	BLAST Identity	GenBank accession
WY-DT1	28S rRNA gene	*Hypochnicium* sp.	99%	KY425696
	ITS	*Hypochnicium* sp.	99%	KP980549
NK-DT1	28S rRNA gene	*Mortierella alpina*	99%	KY773292
	ITS	*Mortierella* sp.	99%	KY779731
